# Transactional sex among men who have sex with men participating in the CohMSM prospective cohort study in West Africa

**DOI:** 10.1371/journal.pone.0217115

**Published:** 2019-11-06

**Authors:** Cheick Haïballa Kounta, Luis Sagaon-Teyssier, Pierre-Julien Coulaud, Marion Mora, Gwenaelle Maradan, Michel Bourrelly, Abdoul Aziz Keita, Stéphane-Alain Babo Yoro, Camille Anoma, Christian Coulibaly, Elias Ter Tiero Dah, Selom Agbomadji, Ephrem Mensah, Adeline Bernier, Clotilde Couderc, Bintou Dembélé Keita, Christian Laurent, Bruno Spire, the CohMSM Study Group

**Affiliations:** 1 Aix Marseille Univ, INSERM, IRD, SESSTIM, Sciences Economiques & Sociales de la Santé & Traitement de l’Information Médicale, Marseille, France; 2 ORS PACA, Observatoire régional de la santé Provence-Alpes-Côte d’Azur, Marseille, France; 3 ARCAD Sida, Bamako, Mali; 4 Espace Confiance, Abidjan, Côte d’Ivoire; 5 Association Africaine Solidarité, Ouagadougou, Burkina Faso; 6 Centre Muraz, Bobo-Dioulasso, Burkina Faso; 7 Espoir Vie Togo, Lomé, Togo; 8 Coalition Internationale Sida, Pantin, France; 9 IRD, INSERM, Univ Montpellier, TransVIHMI, Montpellier, France; British Columbia Centre for Excellence in HIV/AIDS, CANADA

## Abstract

Although the HIV epidemic is generalized in West Africa, some population groups such as men who have sex with men (MSM), especially those engaged in transactional sex (TS), are thought to be particularly more vulnerable to HIV than others. However, few data are available to help identify their health-related needs with a view to implementing targeted prevention interventions. To fill this knowledge gap, we aimed to characterize MSM reporting TS (MSM-TS) and to identify factors associated with their sexual practices using data from the prospective cohort study CohMSM, which was conducted in Burkina Faso, Côte d’Ivoire, Mali and Togo. Three stigmatization sub-scores were constructed (experienced, perceived and internalized). The generalized estimating equation method was used for data analysis. Of the total 630 HIV-negative MSM recruited in CohMSM, 463, 410 and 244 had a follow-up visit at 6, 12 and 18 months, respectively. In a total of 1747 follow-up visits, 478 TS encounters were reported by 289 MSM-TS (45.9%). Of the latter, 91 regularly reported TS (31.5%), 55 (19.0%) stopped reporting TS after baseline, and 53 (18.3%) reported TS after baseline and 90 (31.1%) occasionally reported TS. The following variables, regarding the previous 6 months, were positively associated with TS: being younger (aOR[95%CI]:1.86[1.39–2.50]), less educated (aOR[95%CI]:1.49[1.09–2.03]), unmarried status (aOR[95%CI]:1.79[1.10–2.93]), satisfaction with current sex life (aOR[95%CI]:1.41[1.06–1.88]), group sex with men (aOR[95%CI]:2.07[1.46–2.94]), multiple male sexual partners (aOR[95%CI]:1.85[1.40–2.44]), receptive or versatile anal sex with male partners (aOR [95%CI]:1.48[1.12–1.96]), giving benefits in exchange for sex with a man (aOR[95%CI]:2.80[1.97–3.98]), alcohol consumption (aOR[95%CI]:1.44[1.08–1.93]) and drug use (aOR[95%CI]:1.82[1.24–2.68]) during sex, and finally experiencing stigmatization (aOR [95%CI]:1.15[1.07–1.25]). Condom use during anal sex (aOR[95%CI]:0.73[0.53–0.99]) was negatively associated with TS.

## Introduction

West Africa is a heavily populated region of Sub-Saharan Africa, with an estimated 391 440 million people living in 17 countries [1,2]. Although generalized HIV prevalence among adults in West and Central Africa is 1.5% [1.2–1.9] [3], certain population groups are disproportionately affected by HIV, including men who have sex with men (MSM). For example, prevalence in MSM is estimated at 22% in Togo, 13.7% in Mali, 11.2% in Côte d'Ivoire and 3.6% in Burkina Faso, which is much higher than the respective national prevalences in the general population [3–6]. Despite the high HIV risk in MSM, persistent social barriers in most West African countries means that HIV prevention and care policies continue to focus primarily on the general population [7].

Contrary to what many public health researchers believe, the MSM population is very heterogeneous and comprises different sub-groups such as bisexuals, transgender people, homosexuals and self-identified heterosexual men who have male sexual partners [8–11]. Furthermore, these sub-groups are often strongly associated with specific socio-economic contexts. This heterogeneity may result in different sexual behaviours and consequently, different levels of HIV exposure and transmission risk. Age, educational level, economic disparities and income sources, housing stability, multiple sexual relationships and the exchange of benefits for sex have all been identified in the literature as important factors related to different levels of exposure to risk, in terms of HIV infection and transmission [12–14]. In particular, MSM involved in transactional sex (TS) are thought to be at a much greater risk of acquiring and transmitting HIV than MSM not involved in TS (MSM-NTS) [15–20]. According to UNAIDS, TS is defined as the consensual exchange of sexual services between adults for money or material goods (food, drugs, alcohol, gifts, accommodation or any other benefits), whether regularly or occasionally. This exchange may be explicit (that is to say, a specific tariff for a specific act) or implicit (whereby the tariff is not formally negotiated and compensation may not immediately follow the sexual act) [21]. The main drivers of TS are low financial capacity to support oneself and one’s family [22].

Existing HIV literature on TS has mainly focused on women and shows an association with increased HIV risk [23–30]. Results from studies examining TS in heterosexual men are scarce and often inconclusive [31–33]. With regard to MSM, a systematic review and meta-analysis including data on Sub-Saharan Africa countries, concluded that TS in MSM was associated with significantly higher HIV prevalence [34], especially in Sub-Saharan Africa. This would seem to be explained not only by behavioural factors (e.g., a large number of sexual partners, reduced negotiating power regarding condom use, risky sexual behaviours because of alcohol and drug consumption, etc.), but also by their adverse social and economic context (e.g., low socioeconomic status, limited access to healthcare and to stable housing, as well as multi-layered social stigmatization) [18,5,7,35].

Identifying the health-related needs of MSM involved in TS (whether male sex workers or clients) is important in terms of future HIV prevention policies. One previous study using the same data as this study showed that male clients of male sex workers in West Africa had a lower HIV risk than other MSM in the region who had never been male clients of male sex workers [8]. However, information on MSM receiving benefits (MSM-TS) from these clients remains scarce. To our knowledge, no previous research exists examining the factors associated with TS or the HIV risk in this sub-population. Accordingly, we conducted a longitudinal study using data from CohMSM, a prospective cohort study implemented in MSM in four West African countries (Burkina Faso, Côte d'Ivoire, Mali and Togo), to characterize MSM-TS and to identify factors associated with their sexual practices using Baral et al.’s theoretical framework, which was conceptualized to characterize and analyse five levels (individual, community, network, policy and HIV epidemic stage) of HIV risks in vulnerable populations [36]. Our findings contribute to the literature by offering the first insight into the HIV risk profile of MSM-TS in West Africa and exposure factors associated with this sub-population’s sexual practices.

## Materials and methods

### CohMSM study procedures

In June 2015, the prospective cohort study CohMSM was initiated in four local community-based organisations providing HIV services to MSM in four West African cities: Abidjan (Côte d’Ivoire), Bamako (Mali), Lomé (Togo) and Ouagadougou (Burkina Faso). Its main objectives were to assess both the feasibility and value of implementing a novel three-monthly preventive global care programme for both HIV positive and HIV negative MSM in West Africa, with a view to reducing HIV incidence in this key population, in their female sexual partners and consequently in the general population. Trained peer educators from the four local organisations recruited participants through local MSM networks. Eligibility criteria included being at least 18 years old and reporting at least one anal sexual intercourse (insertive or receptive) with another man in the 3 months preceding study enrolment. Eligible individuals were offered a quarterly preventive global care package which included: i) collection of information on health status, STI symptoms and sexual behaviours, ii) clinical examination, iii) STI diagnosis and treatment if necessary, iv) MSM-specific risk-reduction counselling, and v) condoms and lubricants. At enrolment and at every 6 months of follow-up, participants completed face-to-face interviews with a research assistant who collected information on their sociodemographic and economic characteristics, HIV risk-reduction strategies, alcohol consumption, drug use and stigmatization. Before being included in the cohort, participants systematically received detailed information about the cohort’s objectives and their right to interrupt their participation without justification. Participants had to provide written informed consent. Ethics approval was obtained from the National Ethics Committees of Mali (N°2015/32/CE/FMPOS), Burkina Faso (N°2015-3-037), Côte d’Ivoire (N°021/MSLS/CNER-dkn) and Togo (N°008/2016/MSPSCAB/SG/DPML/ CBRS). The study protocol was designed in accordance with the French National Agency for Research on AIDS and Viral Hepatitis (ANRS) ethical charter for research in developing countries. The ClinicalTrials.gov Identifier is **NCT02626286**. CohMSM is described in detail elsewhere [8].

### Study population

Between 06/2015 and 01/2018, 778 participants were enrolled in CohMSM. As our present study’s aims were to explore HIV risk in MSM-TS and to identify HIV risk exposure factors associated with TS in this sub-population, all HIV-positive participants (n = 148) at all follow-up visits and all related data were excluded from the present analysis. Accordingly, our study focused on the 630 HIV-negative MSM of whom 463, 410 and 244, had a follow-up visit at 6, 12 and 18 months, respectively.

### Variables

#### Outcome

The study outcome was constructed on the basis of the following question: "During the last 6 months, have you been in a situation where you exchanged sex with a man in order to **receive** money, accommodation or any other benefit?". This question was asked at baseline and all follow-up visits. Participants who responded “always” or “sometimes” either at baseline or during follow-up, in contrast to those who responded “never”, were categorized as MSM-TS. The longitudinal nature of our outcome permitted us to identify MSM-TS who regularly practiced TS and those who practised it intermittently. Furthermore, this longitudinal approach ensured that neither MSM-TS nor information on TS-associated variables were lost over time.

#### Explanatory variables

a) **Sociodemographic and socioeconomic characteristics:** sociodemographic characteristics included in the analysis were: age dichotomised at the median (23.7 years); educational level (> = high-school vs. < high-school); stable housing status (stable housing vs. unstable housing); marital status (married or cohabitating vs. single, divorced or widowed). Socioeconomic characteristics were: having an income generating activity (no vs. yes); monthly income dichotomised at the median (50 000 Francs de la Communauté Financière en Afrique, approximately US$ 86.20 in 2019); self-perceived financial situation (comfortable vs. difficult).

b) **Sexual characteristics:** self-defined sexual identity was categorized in a variable as follows: bisexual vs. homosexual/gay vs. transgender; self-defined gender identity was also categorized in another variable as follows: a man exclusively vs. both a man and a woman vs. more a woman than a man; a variable indicating the participant’s current level of sexual satisfaction was divided into three categories as follows: unsatisfactory vs. satisfactory vs. very satisfactory. Dichotomous sexual avoidance variables were constructed indicating whether or not participants practiced HIV risk-reduction strategies (S1 Appendix). Sexual behaviours were recorded using various variables: i) the sexual position taken with male partners was divided into three categories: exclusively insertive vs. receptive or versatile and vs. not documented; ii) the use of condoms and gel during anal sex and condom use during oral sex with male partners, were categorized using yes/no answers; iii) disagreement about condom use with male partners was categorized using a yes/no answer; iv) the number of male sexual partners was dichotomized into none or one *versus* more than one; v) group sex with men was dichotomised using a yes/no answer; vi) alcohol consumption and drug use during sex were dichotomized using yes/no answers; vii) sudden sexual violence by male partners was dichotomised into yes or no; viii) giving benefits in exchange for sex with a man was dichotomised into yes or no; and ix) having had at least one sexually transmitted infection (STI) was dichotomised into yes or no.

The information provided by all the variables listed above concerned the 6 months before the survey, but another variable, ‘searching for male sexual partners on the internet’, which was also dichotomised into yes or no, concerned the previous 4 weeks. All these HIV risk behaviour variables are similar to those used in other studies [37,9,20].

c) **Homosexuality-related stigmatization during the previous 6 months**: we constructed the following three scales for homosexuality-related stigmatization, each scale ranging from 0 to 10, based on items from a previous study (S2 Appendix) [38]: “experienced homosexual stigmatization” (based on 5 items, Cronbach’s alpha = 0.83); “perceived homosexual stigmatization” (based on 11 items, Cronbach’s alpha = 0.54); and “internalized homosexual stigmatization” (based on 8 items, Cronbach’s alpha = 0.73).

### Statistical analysis

Descriptive analysis was conducted to compare baseline sociodemographic and socioeconomic characteristics, and to compare sexual behaviours between MSM-TS and MSM not practising TS (MSM-NTS). Categorical variables were compared between the two groups using Pearson's chi-squared test (χ2).

To identify TS associated factors, univariate and multivariate analyses were then performed using the generalized estimating equation (GEE) model which offers population-averaged estimates while controlling for the correlation over time of repeated measures for the same individual. Given the large number of variables, only independent variables with a p < 0.05 in univariate logistic regression were retained in the multivariate model. Sociodemographic and socioeconomic variables were time-fixed as related information was only collected at baseline. In contrast, questions regarding sexual behaviour and stigmatization variables were asked at every follow-up visit and were therefore defined as time-varying.

The final multivariate model was obtained using a backward elimination procedure based on the quasi-likelihood Akaike’s information criterion (QAIC) [39] and global p-values (type III). Fixed effects for each study country were specified in order to avoid bias arising from differences in sample sizes. All statistical analyses were performed using Stata version 13.0 (StataCorp, College Station, Texas, USA).

## Results

### Overall sample description

Of the 778 MSM enrolled in CohMSM study, 148 (19.0%) were HIV-positive and were excluded from the present analysis. Baseline characteristics for included and excluded participants are presented in the Supplementary Table (S3 Appendix). Excluded participants were more likely to self-define their gender as both a man and a woman than included participants. No other significant difference was found between the two groups.

In total, 630 MSM were HIV-negative over all follow-up visits, and all were included in the present analysis. In a total of 1747 visits, 289 MSM-TS (45.9% of the 630 study participants) reported 478 TS encounters. Ninety-one of these MSM-TS regularly reported TS (31.5%), 55 (19.0%) stopped reporting TS after baseline, 53 (18.3%) reported TS after baseline and 90 (31.1%) occasionally reported TS.

The baseline comparative analysis between 289 MSM-TS (45.9%) and 341 MSM-NTS (54.1%) revealed significant differences with respect to sociodemographic characteristics and sexual behaviours (**Table 1**). More specifically, a significantly higher proportion of MSM-TS was found for the following: i) unmarried status (single, divorced or widowed) 80.3% vs. 67.4%, p = 0.001), ii) self-defined gender as both a man and a woman (47.9% vs. 39.3%, p = 0.030), iii) having anal sex (receptive or versatile) with male sexual partners (68.2% vs. 56.3%, p = 0.008), and iv) giving benefits in exchange for sex with a man (15.2% vs. 8.5%, p = 0.009).

**Table 1:** Comparative analysis of the baseline characteristics of the study sample (n = 630).

		MSM-TS			MSM-NTS		
Sociodemographic and socioeconomic characteristics		n = 289 (45.9%)			n = 341 (54.1%)		^*a*^p Value
		n (%)			n (%)		
Follow-up visit							
At baseline			199 (41.6)		431 (34.0)		**<0.001**
At 6 months			96 (20.1)		367 (28.9)		
At 12 months			103 (21.6)		307 (24.2)		
At 18 months			80 (16.7)		164 (12.9)		
Study country (n = 630)							
Mali			150 (51.9)		99 (29.0)		**<0.001**
Cote d'Ivoire			51 (17.7)		84 (24.6)		
Burkina			41 (14.2)		88 (25.8)		
Togo			47 (16.3)		70 (20.5)		
Age group relative to the median (n = 630)							
Median [IQR]			23.2 [4.1]		23.9 [4.6]		
> = 23.7 years			114 (39.4)		189 (55.4)		**<0.001**
< 23.7 years			175 (60.6)		152 (44.6)		
Educational level (n = 630)							
≥ high-school diploma			80 (27.7)		134 (39.3)		**<0.001**
< high-school diploma			182 (63.0)		161 (47.2)		
ND^b^			27 (9.3)		46 (13.5)		
Marital status (n = 630)							
Married or living in a couple			30 (10.4)		65 (19.1)		**0.001**
Single, Divorced, Widowed			232 (80.3)		230 (67.4)		
ND^b^			27 (9.3)		46 (13.5)		
Had an income generating activity (n = 630)							
No			207 (71.6)		231 (67.7)		0.291
Yes			82 (28.4)		110 (32.3)		
Monthly income relative to the median (n = 630)							
Median [IQR]			50000 [30000]		55000 [25000]		
< = 50 000 Fcfa			149 (51.6)		165 (48.4)		0.473
> 50 000 Fcfa			106 (36.7)		125 (36.7)		
ND^b^			34 (11.7)		51 (14.9)		
Financial perception (n = 630)							
Comfortable			76 (26.3)		103 (30.2)		0.088
Difficult			186 (64.4)		192 (56.3)		
ND^b^			27 (9.3)		46 (13.5)		
Stable housing (n = 630)							
No			65 (22.5)		90 (26.4)		0.087
Yes			197 (68.2)		205 (60.1)		
ND^b^			27 (9.3)		46 (13.5)		
Self-defined sexual identity (n = 630)							
Bisexual			163 (56.4)		184 (54.0)		0.539
Homosexual/Gay			126 (43.6)		157 (46.0)		
Transgender			0 (0)		0 (0)		
Self-defined gender identity (n = 630)							
Man exclusively			150 (52.1)		207 (60.7)		**0.030**
Both a man and woman			138 (47.9)		134 (39.3)		
More a woman than a man			0 (0)		0 (0)		
Sexual positioning with male partners in the previous 6 months (n = 630)							
Exclusively insertive			85 (29.4)		141 (41.4)		**0.008**
Receptive or versatile			197 (68.2)		192 (56.3)		
ND^b^			7 (2.4)		8 (2.4)		
Had given benefits in exchange for sex with a man (n = 630)							
No			245 (84.8)		312 (91.5)		**0.009**
Yes			44 (15.2)		29 (8.5)		

^a^p Calculated with Pearson’s chi-squared test (χ2) for categorical variables, Student’s t-test for continuous variables.

^b^ND = not documented. Includes missing data, “does not know” and “no response” terms. This category was introduced in order not to lose observations because of missing values.

Age [mean ± standard deviation] = MSM-TS [24.7±4.4] vs. MSM-NTS [25.9±5.9]

Monthly income [mean ± standard deviation] = MSM-TS [54000±5.7] vs. MSM-NTS [58000±8.9]

A significantly lower proportion of MSM-TS had a high school diploma (27.7% MSM-TS versus 39.3% MSM-NTS, p<0.001). Other differences observed were that MSM-TS were younger (median [IQR]:23.2 [4.1]) (median [IQR]:23.9 years [4.6]) (p<0.001) and that despite being less likely to have work, MSM-TS tended to have stable housing (68.2% vs. 60.1%, p = 0.087).

However, no significant difference was found between the two groups for income generating activity, monthly income or self-defined sexual identity.

### Results from multivariate analysis

In a total of 1747 visits, 1269 corresponded to MSM-NTS (median [IQR] follow-up time: 12.4 [12.2] months) and 478 visits corresponded to MSM-TS (median [IQR] follow-up time: 12.1 [12.1] months). Results from the multivariate analysis of TS-associated factors (Table 2), indicated that younger MSM were significantly more likely to practice TS [adjusted odds ratio (aOR) and 95% confidence interval (95% CI):1.86(1.39–2.50)]. In addition, participants who had an educational level <high-school diploma (aOR[95%CI]:1.49[1.09–2.03]) and who were unmarried (single, divorced or widowed) (aOR[95%CI]:1.79[1.10–2.93]) were significantly more likely to be MSM-TS.

**Table 2:** Factors associated with transactional sex among men who have sex with men in West Africa: Univariate and multivariate analyses using the generalized estimating equation (n = 630, 1747 follow-up visits).

Background characteristics		Follow- up visits				Univariate analysis^*a*^			Multivariate analysis^*b*^	
		MSM-TS		MSM-NTS						
		n = 478 (100%)		n = 1 269 (100%)		OR [95% CI]^*c*^	p^*e*^		aOR [95% CI]^d^	p^*e*^
Median [IQR] follow-up time	12.1 [12.1]			12.4 [12.2]						
Follow-up time-point (N = 1747)										
baseline		199 (41.6)		431 (34.0)		Ref			Ref	
6 months		96 (20.1)		367 (28.9)		0.57 [0.45–0.72]			0.68 [0.50–0.92]	
12 months		103 (21.6)		307 (24.2)		0.75 [0.59–0.95]	**<0.001**		0.90 [0.66–1.23]	**0.001**
18 months		80 (16.7)		164 (12.9)		1.04 [0.79–1.36]			1.38 [0.97–1.96]	
Study country										
Mali		277 (58.0)		493 (38.9)		Ref			Ref	
Cote d'Ivoire		71 (14.8)		272 (21.4)		0.48 [0.33–0.70]			0.43 [0.28–0.68]	
Burkina		68 (14.2)		273 (21.5)		0.47 [0.32–0.68]	**<0.001**		0.31 [0.20–0.46]	**<0.001**
Togo		62 (13.0)		231 (18.2)		0.50 [0.34–0.73]			0.50 [0.33–0.76]	
Age group relative to the median										
> = 23.7 years		181 (37.9)		676 (53.3)		Ref			Ref	
< 23.7 years		297 (62.1)		593 (46.7)		1.88 [1.43–2.48]	**<0.001**		1.86 [1.39–2.50]	**<0.001**
Educational level										
≥ high-school diploma		133 (27.8)		506 (39.9)		Ref			Ref	
< high-school diploma		317 (66.3)		711 (56.0)		1.73 [1.28–2.34]	**<0.001**		1.49 [1.09–2.03]	**0.006**
ND^f^		28 (5.9)		52 (4.1)						
Marital status										
Married or living in a couple		45 (9.4)		217 (17.1)		Ref			Ref	
Single, Divorced, Widowed		405 (84.7)		1000 (78.8)		1.87 [1.22–2.88]	**0.007**		1.79 [1.10–2.93]	**0.020**
ND^f^		28 (5.9)		52 (4.1)						
Monthly income relative to the median										
< = 50 000 Fcfa		253 (52.9)		677 (53.3)		Ref				
> 50 000 Fcfa		186 (38.9)		511 (40.3)		1.01 [0.76–1.35]	0.935			
ND^f^		39 (8.2)		81 (6.4)						
Had an income generating activity										
No		345 (72.2)		824 (64.9)		Ref				
Yes		133 (27.8)		445 (35.1)		0.73 [0.54–0.98]	**0.035**			
Had given benefits in exchange for sex with a man										
No		384 (80.3)		1193 (94.0)		Ref			Ref	
Yes		94 (19.7)		76 (6.0)		3.25 [2.39–4.43]	**<0.001**		2.80 [1.97–3.98]	**<0.001**
Stable housing										
No		116 (24.3)		342 (27.0)		Ref				
Yes		334 (69.9)		875 (68.9)		1.14 [0.83–1.56]	0.435			
ND^f^		28 (5.8)		52 (4.1)						
Self-defined gender identity										
Man exclusively		239 (50.1)		799 (63.0)		Ref				
Both a man and woman		238 (49.9)		469 (37.0)		1.62 [1.29–2.04]	**<0.001**			
Qualification of current sex life										
Satisfactory		364 (76.1)		1063 (83.8)		Ref			Ref	
Very satisfactory		114 (23.9)		206 (7(16.2)		1.53 [1.19–1.95]	**<0.001**		1.41 [1.06–1.88]	**0.020**
Had a female partner during the previous 6 months										
No		256 (53.6)		638 (50.3)		Ref				
Yes		222 (46.4)		631 (49.7)		1.03 [0.82–1.28]	0.826			
Had at least one STI during lifetime										
No		434 (91)		1107 (87.9)		Ref				
Yes		43 (9.0)		152 (12.1)		0.79 [0.51–1.22]	0.285			
Sexual positioning with male partners in the previous 6 months										
Exclusively insertive		148 (31.0)		518 (40.8)		Ref			Ref	
Receptive or versatile		314 (65.7)		647 (51.0)		1.51 [1.17–1.94]	**<0.001**		1.48 [1.12–1.96]	**0.012**
ND^f^		16 (3.3)		104 (8.2)						
Condom use with male partners during anal sex in the previous 6 months										
No		91 (19.0)		174 (13.7)		Ref			Ref	
Yes		387 (81.0)		1095 (86.3)		0.72 [0.55–0.94]	**0.016**		0.73 [0.53–0.99]	**0.049**
Condom use with male partners during oral sex in the previous 6 months										
No		345 (72.2)		760 (59.9)		Ref				
Yes		133 (27.8)		509 (40.1)		0.67 [0.53–0.84]	**0.001**			
Gel use with male partners during anal sex in the previous 6 months										
No		243 (50.8)		543 (42.8)		Ref				
Yes		235 (49.2)		726 (57.2)		0.72 [0.59–0.89]	**0.003**			
Disagreement about condom use with male partners in the previous 6 months										
No		375 (78.4)		1114 (87.8)		Ref				
Yes		103 (21.6)		155 (12.2)		1.74 [1.33–2.27]	**<0.001**			
Alcohol consumption during sex in the previous 6 months										
No		8 (1.7)		53 (4.2)		Ref			Ref	
Yes		91 (19.0)		170 (13.4)		1.31 [1.02–1.69]	**0.027**		1.44 [1.08–1.93]	**0.047**
ND^f^		379 (79.3)		1046 (82.4)						
Drug use during sex in the previous 6 months										
No		366 (76.6)		1097 (86.5)		Ref			Ref	
Yes		70 (14.6)		92 (7.2)		2.00 [1.43–2.81]	**<0.001**		1.82 [1.24–2.68]	**0.010**
ND^f^		42 (8.8)		80 (6.3)						
Sudden sexual violence by male partners in the previous 6 months										
No		433 (90.6)		1215 (95.7)		Ref				
Yes		45 (9.4)		54 (4.3)		1.92 [1.28–2.87]	**0.002**			
Number of male sexual partners in the previous 6 months										
< = One		103 (21.6)		491 (38.7)		Ref			Ref	
More than one		375 (78.4)		778 (61.3)		1.99 [1.56–2.54]	**<0.001**		1.85 [1.40–2.44]	**<0.001**
Searched for male sexual partners on the internet in the previous 4 weeks										
No		240 (50.2)		798 (62.9)		Ref				
Yes		238 (49.8)		471 (37.1)		1.41 [1.14–1.75]	**0.001**			
Group sex with men										
No		377 (78.9)		1151 (90.8)		Ref			Ref	
Yes		101 (21.1)		117 (9.2)		2.35 [1.77–3.10]	**<0.001**		2.07 [1.46–2.94]	**<0.001**
**HIV risk-reduction strategies practiced**										
Limited the number of sexual partners										
No		172 (36.0)		399 (31.8)		Ref				
Yes		306 (64.0)		870 (68.2)		0.82 [0.69–1.02]	0.077			
Avoided sexual relations when drunk or when consuming other psychoactive products in order to reduce the risk of HIV infection										
No		85 (17.8)		131 (10.4)		Ref				
Yes		393 (82.2)		1138 (89.6)		0.67 [0.50–0.90]	**0.008**			
**Stigmatisation Scores**										
Experienced stigmatisation in the previous 6 months		478 (100)		1269 (100)		1.16 [1.10–1.25]	**<0.001**		1.15 [1.07–1.25]	**<0.001**

^a^Univariate analysis using a generalized estimating equation.

^b^Multivariate analysis using a multivariate stepwise generalized estimating equation.

^c^OR = odds ratio; IC = confidence interval.

^d^aOR = adjusted odds ratio; IC = confidence interval.

^e^p Calculated with Wald chi2 test.

^f^ND = not documented. Includes missing data, “does not know” and “no response” terms. This category was introduced in order to not lose observations because of the missing values. Their odds ratios were estimated but are not presented in Table 2.

With respect to sexual behaviours (measured in terms of the previous 6 months), participants who gave benefits in exchange for sex with a man (aOR[95%CI]:2.80[1.97–3.98]), those who had multiple male sexual partners (aOR[95%CI]:1.85[1.40–2.44]), those who practised receptive or versatile anal sex with male sexual partners (aOR[95%CI]:1.48[1.12–1.96]), those who had group sex with men (aOR[95%CI]:2.07[1.46–2.94]), and those who reported alcohol consumption during sex (aOR[95%CI]:1.44[1.08–1.93]) and/or drug use during sex (aOR[95%CI]:1.82[1.24–2.68]), were all significantly more likely to practice TS. In addition, participants who were very satisfied with their current sex life (aOR[95%CI]:1.41[1.06–1.88]) were also significantly more likely to practice TS. In contrast, participants who reported condom use during anal sex in the previous 6 months (aOR[95%CI]:1.359[0.99–1.86]; p = 0.054) were significantly less likely to practice TS.

Furthermore, the more MSM had experienced stigmatization in the previous 6 months the more likely they were to practice TS (aOR [95%CI]:1.15[1.07–1.25]).

Finally, at 6 months of follow-up, a 68.9% decrease was observed in reported TS with respect to baseline (aOR[95%CI]:0.68[0.50–0.92]).

## Discussion

Our results showed that 289 MSM (45.9%) included in our study sample had received benefits for transactional sex (TS) (MSM-TS) with other men. With respect to MSM not practising TS (MSM-NTS), these MSM-TS had high-risk HIV exposure practices as well as socioeconomic difficulties. Although our study sample is not representative of the whole MSM population in West Africa, the high proportion of MSM-TS which we found reflects findings in the literature concerning TS among MSM in other countries [9,20]. Overall, we observed various TS tendencies according to loss to follow-up over time during the cohort. More specifically, we found a significant decrease in the probability of TS at 6 months of follow-up, but a tendency towards an increased probability at 18 months. In order to analyse factors associated with TS, we were inspired by the first two HIV vulnerability levels (individual and community) of Baral et al.’s modified social ecological model (2013) which correspond to our TS-associated factors [36]. This analytical tool was considered a relevant theoretical framework by another study to analyse multilevel vulnerability to HIV in a sub-population of MSM-TS [20]. Our results show that younger, low-educated participants were significantly more likely to practice TS. These results are consistent with those of another study which analysed the associations between HIV risky sexual behaviours and MSM-TS in Latin America [15]. These two factors—age and educational level–may increase HIV vulnerability in MSM with less sexual experience and those who have more difficulty accessing information on safe sexual behaviours in TS relationships. Consequently, improving school retention programmes and education policies for young people, especially MSM, are needed in the West African context. School authorities in the region could experiment with conditional cash transfer programmes. By improving young people’s economic situation, these prevention interventions could help to both keep students in school and boost safe sex. This would lead to much less dependence on TS and therefore a lower HIV risk [40]. Our results also show that unmarried participants were significantly more likely to practice TS. One possible reason for this is that being unmarried and living alone in the African context—where stigma and discrimination are very present, may limit economic opportunities for MSM, and may drive them to keep their sexual activities “hidden” [41].

Multivariate estimation did not show any association between TS and self-defined gender identity, although a significant difference was observed between MSM-TS and MSM-NTS in the univariate model. Although studies have shown that gender nonconforming MSM or MSM who display feminine characteristics experience more mental distress than their gender conforming counterparts [42,43], no such association with TS practice has been shown in West Africa. In-depth studies focused on acquiring a greater understanding of these gender identities will shed light on their links with HIV transmission.

Furthermore, our results highlighted that participants practicing condomless anal sex and those very satisfied with their current sex life were more likely to practice TS. In addition, MSM who reported receptive sex or a versatile sexual position during anal intercourse were significantly more likely to practice TS. These practices (receptive sex and condomless anal sex) constitute the greatest HIV transmission risk in MSM, particularly MSM-TS [44,45]. Our study did not collect data on the reasons for sexual satisfaction. However, another study showed that MSM find unprotected sexual intercourse to be more satisfying, and that they feel that condom use increases sexual discomfort [46]. This may explain both the high level of sexual satisfaction in our MSM-TS and the high level of reported condomless anal sex.

Our results also highlight that those consuming alcohol and/or using drugs during sex were significantly more likely to practice TS. Two possible reasons for this are the burden of stigmatization of their sexual practices and the search for strong sexual sensations [47] although our study design prevented us from verifying these. Indeed, studies in different contexts have shown an association between psychoactive substance use and increased risky HIV behaviours, such as unprotected receptive anal sex, among MSM, and particularly in MSM-TS [48–52]. We believe that risk-reduction interventions focusing on substance use are necessary to mitigate the HIV epidemic in this population.

Participants who reported multiple male sexual partners during the previous 6 months, and group sex with men, were significantly more likely to practice TS. This finding is consistent with other studies showing that the larger the sexual network of MSM, the greater the probability of TS with a member who already practices it, as MSM tend to use their networks to find male sexual partners [53]. Consequently, there is a greater probability of being exposed to HIV-positive partners who do not practice HIV prevention measures [54]. Moreover, some studies have demonstrated multiple concurrent heterosexual partnerships and little or no condom use within TS partnerships in Sub-Saharan Africa [55,23,13]. Accordingly, we recommend risk-reduction strategies that not only include components aimed at reducing relationships with multiple and concurrent sexual partners, but also include negotiation and communication skills aimed at encouraging systematic condom use among MSM. Furthermore, our results showed that monthly incomes of MSM-TS (86.20 US$) were very low compared with the GDP per capita (US$) of Sub-Saharan Africa (3500 US$ for 2016). This confirms the low economic status of MSM in general and the wealth inequalities in their community [56]. In this context, young MSM may enter various sexual partnerships, often concurrently, because of multiple financial needs. It is therefore necessary to use a social and ecological model to understand MSM motivations for TS, with a view to optimizing related social, behavioural and structural interventions.

Our results also showed that those giving benefits in exchange for sex with a man were significantly more likely to practice TS. This implies an overlap in the proportion of those who both receive and give benefits in the TS context. These findings however contrast with prior research on MSM in Tanzania, Kenya and US [57–59], and seem to be based on a pattern wherein financial and material benefits play the role of facilitators, with MSM receiving benefits for sex finding themselves in situations where they would otherwise not choose to have sex with a particular partner, and giving benefits in situations where the partner does not find them attractive. Future studies, especially qualitative ones, should examine whether this group (i.e., those who both give and receive benefits in TS with men) has specific identifying characteristics and behavioural risks, in terms of sexual identity, partnership structure, and health. Knowledge of these characteristics and potentially associated risks could contribute to creating effective tailored health interventions for this group.

Finally, our study showed that those who had experienced stigmatization were significantly more likely to practice TS. To measure stigmatization, we decided to use the Homosexuality-Related Stigma Scale, developed and validated in Vietnam [38]. This scale takes into account all three types of MSM stigmatization (experienced, perceived and internalized). The simple questions used in the scale’s questionnaire allowed us to test for associations between TS and each stigmatization type. Analyses yielded significant results, proving that stigma is indeed a profound problem in this population. This confirms the value of performing more in-depth studies to validate this scale among MSM in West Africa. Social norms and the fear of being stigmatized may constitute barriers to finding regular sex partners, which in turn may push them to engage more in TS [60,11,61]. Although our results do not provide a reason as to why MSM-TS were more likely to be stigmatized than MSM-NTS, it is possible that MSM-TS reveal their homosexual orientation more often when looking for a client, thereby increasing the risk of being stigmatized by the general public. Importantly, the level of stigma experienced by MSM-TS may limit their use of healthcare services. This has been shown in prior research highlighting that access to and utilization of HIV prevention and care by the MSM population is influenced by certain social vulnerabilities such as stigmatization [54,62,63]. Our results suggest the need for stigmatization mitigation interventions to optimize MSM-TS linkage to HIV prevention and treatment services in West Africa.

The primary strengths of our study come from the fact that the CohMSM study was performed in four different West African countries, was longitudinal in nature, and had four scheduled follow-up visits over 18 months. Some study limitations should be taken into account when interpreting our results. First, we were not able to investigate our participants' motivations for practising TS (i.e., out of financial necessity or for pleasure), or indeed whether they identified themselves as a sex worker or not. Second, given the declarative nature of the data and the fact that respondents participated in face-to-face interviews, social desirability bias is possible. Accordingly, sexual risk behaviours may have been underreported. However, this bias was perhaps minimized by the fact that the research assistants involved all worked close to the ground, came from recognized non-governmental organizations, and were directly involved with the MSM population. Furthermore, as participating MSM had a follow-up visit every 3 months, it is likely that a trustful relationship emerged over time with the research assistants, and consequently social desirability bias was further reduced. A third limitation is that we used stigmatization variables defined in the Homosexuality-Related Stigma Scale, developed and validated in Vietnam, but not previously used or validated in West Africa.

Despite these limitations, our results may provide information useful for the optimization of prevention interventions for this this vulnerable MSM sub-group in West Africa. Biomedical, behavioural, and structural interventions such as the implementation of early antiretroviral therapy and pre-exposure prophylaxis, as well as interventions to reduce stigmatization, are urgently needed to mitigate the effect of the continued HIV epidemic in this region.

## Conclusion

Little is known about MSM-TS in West Africa. The high proportion of MSM-TS found in our study, characterized by socioeconomic difficulties and risky HIV exposure behaviours, underlines the need for greater attention to be paid to this population. Our results also show the importance of HIV prevention interventions in this sub-population, and underline the need to develop more effective targeted prevention interventions at the community level (especially concerning the fight against stigma), as well as interventions addressing individual factors (pre-exposure prophylaxis, treatment as prevention and post-exposure prophylaxis). More in-depth representative multicentre research targeting MSM-TS is needed to better understand the multifaceted and multilevel factors associated with TS among the MSM population in West Africa, in order to take account behavioural heterogeneity in this population, and to classify inter-country similarities and differences.

## Supporting information

S1 Appendix Variables of HIV risk-reduction strategies. (DOCX)

S2 Appendix Variables used to construct stigmatization scores. (DOCX)

S3 Appendix Comparative analysis of the baseline characteristics of included versus excluded participants in our study. (DOCX)

S4 Appendix Data set names and variables. (DOCX)
