# Supplementary material for: Transactional sex among men who have sex with men participating in the CohMSM prospective cohort study in West Africa
Source: PLoS One. 2019 Nov 6;14(11):e0217115. doi: 10.1371/journal.pone.0217115 (PMC6834336; doi:10.1371/journal.pone.0217115)
Supplement: S3 Appendix — (DOCX) [file pone.0217115.s003.docx]

**S3 Appendix:** Comparative analysis of the baseline characteristics of included versus excluded participants in our study (CohMSM study sample N=778).

| Sociodemographic and socioeconomic characteristics |  | Participants included  n=630 (81%)  n (%) |  | Participants excluded  n=148 (19%)  n (%) |  | ***^a^***p Value |
| --- | --- | --- | --- | --- | --- | --- |
|  |  |  |  |  |  |  |
|  |  |  |  |  |  |  |
| Study country (n=778) | | | | | | |
| Mali |  | 249 (39.5) |  | 64 (43.3) |  | 0.065 |
| Cote d'Ivoire |  | 135 (21.4) |  | 42 (28.5) |  |  |
| Burkina |  | 129 (20.5) |  | 25 (16.7) |  |  |
| Togo |  | 117 (18.6) |  | 17 (11.5) |  |  |
| Age group relative to the median (n=778) | | | | | | |
| Median [IQR] |  | 23.6 [4.4] |  | 23.3 [4.2] |  | 0.146 |
| >= 23.7 years |  | 303 (48.1) |  | 81 (54.7) |  |  |
| < 23.7 years |  | 327 (51.9) |  | 67 (45.3) |  |  |
| Educational level (n=778) | | | | | | |
| ≥ high-school diploma |  | 214 (34.0) |  | 59 (39.9) |  | 0.339 |
| < high-school diploma  ND^b^ |  | 343 (54.4)  73 (11.6) |  | 71 (47.9)  18 (12.2) |  |  |
| Marital status (n=778) | | | | | | |
| Married or living in a couple |  | 95 (15.1) |  | 30 (20.3) |  | 0.292 |
| Single, Divorced, Widowed  ND^b^ |  | 462 (73.3)  73 (11.6) |  | 103 (69.6)  15 (10.1) |  |  |
| Had an income generating activity (n=778) | | | | | | |
| No |  | 438 (69.5) |  | 105 (70.9) |  | 0.734 |
| Yes |  | 192 (30.5) |  | 43 (29.1) |  |  |
| Monthly income relative to the median ((n=778) | | | | | | |
| Median [IQR] |  | 52500 [27500] |  | 55000 [30000] |  | 0.993 |
| <= 50 000 Fcfa |  | 314 (49.8) |  | 73 (49.3) |  |  |
| > 50 000 Fcfa  ND^b^ |  | 231 (36.7)  85 (13.5) |  | 55 (37.2)  20 (13.5) |  |  |
| Financial perception (n=778) | | | | | | |
| Comfortable |  | 179 (28.4) |  | 46 (31.1) |  | 0.465 |
| Difficult  ND^b^ |  | 378 (60.0)  73 (11.6) |  | 81 (54.7)  21 (14.2) |  |  |
| Stable housing (n=778) | | | | | | |
| No |  | 155 (24.6) |  | 39 (26.4) |  | 0.679 |
| Yes  ND^b^ |  | 402 (63.8)  73 (11.6) |  | 89 (60)  20 (13.6) |  |  |
| Self-defined sexual identity (n=778) | | | | | | |
| Bisexual |  | 347 (55.1) |  | 81 (54.7) |  | 0.939 |
| Homosexual/Gay |  | 283 (44.9) |  | 67 (45.3) |  |  |
| Transgender |  | 0 (0) |  | 0 (0) |  |  |
| Self-defined gender identity (n=778) | | | | | | |
| Man exclusively |  | 358 (56.7) |  | 65 (43.9) |  | **0.005** |
| Both a man and woman |  | 272 (43.3) |  | 83 (56.1) |  |  |
| More a woman than a man |  | 0 (0) |  | 0 (0) |  |  |
| Sexual positioning with male partners in the previous 6 months (n=778) | | | | | | |
| Exclusively insertive |  | 226 (35.9) |  | 46 (31.1) |  | 0.330 |
| Receptive or versatile  ND^b^ |  | 389 (61.7)  15 (2.4) |  | 96 (64.9)  6 (4.0) |  |  |
| Had given benefits in exchange for sex with a man (n=778) | | | | | | |
| No |  | 557 (88.4) |  | 136 (91.9) |  | 0.222 |
| Yes |  | 73 (11.6) |  | 12 (8.1) |  |  |

^a^p Calculated with Pearson’s chi-squared test (χ2) for categorical variables, Student’s t-test for continuous variables.
