# Supplementary material for: Transactional sex among men who have sex with men participating in the CohMSM prospective cohort study in West Africa
Source: PLoS One. 2019 Nov 6;14(11):e0217115. doi: 10.1371/journal.pone.0217115 (PMC6834336; doi:10.1371/journal.pone.0217115)
Supplement: S4 Appendix — (DOCX) [file pone.0217115.s004.docx]

**S4 Appendix:** Data set names and variables

**Data set name**: Transactional sex data set

| **Variables names** | **Label** |
| --- | --- |
| questio | Follow-up visit |
| pays | Study country |
| recevoir_cc | Outcome1 |
| recevoir_c | Outcome2 |
| age_med_c | Age |
| q002_miss_c | Educational level |
| statut_mat_c | Marital status |
| revenu_c | Monthly income |
| activite_c | Had an income generating activity |
| q013_c | Stable housing |
| perceptionf_r2 | Financial perception |
| donner_c | Had given benefits in exchange for sex with a man |
| position_sexuelle | Sexual positioning with male partners in the previous 6 months |
| ident_sexuelle_c | Self-defined sexual identity |
| q119_c | Self-defined gender identity |
| condom_c | Condom use with male partners during anal sex in the previous 6 months |
| condom_oral_c | Condom use with male partners during oral sex in the previous 6 months |
| gel_c | Gel use with male partners during anal sex in the previous 6 months |
| femme | Had a female partner during the previous 6 months |
| min_1_ist_c | Had at least one STI during lifetime |
| q079_096 | Sudden sexual violence by male partners in the previous 6 months |
| q117_c | Qualification of current sex life |
| alcool_dicho_miss | Alcohol consumption during sex in the previous 6 months |
| drogue_6mois_miss_c | Drug use during sex in the previous 6 months |
| q077_094 | Disagreement about condom use with male partners in the previous 6 months |
| q031_1 | Had a casual female partner during the previous 6 months |
| q027_c | Number of male sexual partners in the previous 6 months |
| internet | Searched for male sexual partners on the internet in the previous 4 weeks |
| groupe_c | Group sex with men |
| q116a_c | HIV risk-reduction strategies practiced |
| q116b_c | HIV risk-reduction strategies practiced |
| q116c_c | HIV risk-reduction strategies practiced |
| q116d_c | HIV risk-reduction strategies practiced |
| q116e_c | HIV risk-reduction strategies practiced |
| q116f_c | HIV risk-reduction strategies practiced |
| q116g_c | HIV risk-reduction strategies practiced |
| q116h_c | HIV risk-reduction strategies practiced |
| q116i_c | HIV risk-reduction strategies practiced |
| q116j_c | HIV risk-reduction strategies practiced |
| q116k_c | HIV risk-reduction strategies practiced |
| q116l_c | HIV risk-reduction strategies practiced |
| q116m_c | HIV risk-reduction strategies practiced |
| q116n_c | HIV risk-reduction strategies practiced |
| q116o_c | HIV risk-reduction strategies practiced |
| q116p_c | HIV risk-reduction strategies practiced |
| stigma_6mois | Experienced stigmatisation in the previous 6 months |
| stigma_percu | Perceived stigmatisation in the previous 6 months |
| stigma_interieur | Internalized stigmatisation in the previous 6 months |
